# Self-eating and Heart: The Emerging Roles of Autophagy in Calcific Aortic Valve Disease

**DOI:** 10.14336/AD.2021.0101

**Published:** 2021-08-01

**Authors:** Yunlong Fan, Jiakang Shao, Shixiong Wei, Chao Song, Yanan Li, Shengli Jiang

**Affiliations:** ^1^Medical School of Chinese PLA, Beijing 100853, China.; ^2^Department of Cardiovascular Surgery, the First Medical Centre of Chinese PLA General Hospital, Beijing 100853, China

**Keywords:** autophagy, heart aging, cell death, calcific aortic valve disease, calcification

## Abstract

Autophagy is a self-degradative pathway by which subcellular elements are broken down intracellularly to maintain cellular homeostasis. Cardiac autophagy commonly decreases with aging and is accompanied by the accumulation of misfolded proteins and dysfunctional organelles, which are undesirable to the cell. Reduction of autophagy over time leads to aging-related cardiac dysfunction and is inversely related to longevity. However, despite the increasing interest in autophagy in cardiac diseases and aging, the process remains an undervalued and disregarded object in calcific valvular disease. Neither the nature through which autophagy is triggered nor the interplay between autophagic machinery and targeted molecules during aortic valve calcification are fully understood. Recently, the upregulation of autophagy has been shown to result in cardioprotective effects against cell death as well as its origin. Here, we review the evidence that shows how autophagy can be both beneficial and detrimental as it pertains to aortic valve calcification in the heart.

Calciﬁc aortic valve disease (CAVD) is the most prevalent valvular disease in the elderly and has become a rising social and health burden, especially in developed countries that have an aging baby boomer generation [1]. Once CAVD occurs, it unavoidably moves ahead and has a poor outcome in symptomatic patients. Unfortunately, no pharmacological treatments have proven effective in preventing or reversing disease progression. When the disease becomes severe and symptomatic, surgical or transcatheter aortic valve replacement is the only effective cure, with which not all patients can be fitted [2]. Therefore, novel pharmaceuticals targeting CAVD are currently unmet clinical needs.

Although not completely the same, CAVD and atherosclerosis show similarities in clinical risk factors, including male sex, smoking habits, hypertension, dyslipidemia, and diabetes [3] and share common pathological processes associated with endothelial damage, lipoprotein deposition, chronic inflammation, and matrix remodeling [4]. Historically, CAVD manifests significantly in progressively thickened, fibrosed, and calcified valve cusps. Similar to atherosclerotic lesions, these early valve lesions are ignited by endothelial damage, with lipid accumulation and subsequent oxidation that result in inflammatory infiltration within the endothelium layer, which contains macrophages, T cells, and mast cells that release proinflammatory cytokines. Over the years, prolonged “wear and tear” stimuli lead to fibrocalcific remodeling and osteogenic calcification, akin to new bone formation [5].

During the cell life cycle, autophagy constitutes a cell survival mechanism that counteracts harmful stimuli, including extracellular (e.g., hypoxia, nutrient deprivation, and inflammation) and intracellular (e.g., endoplasmic reticulum stress [ERS], reactive oxygen species [ROS], and damaged mitochondria) damage [6, 7]. Basal autophagy maintains proper physiological function by regulating cellular homeostasis, metabolic balance, and protein structural integrity. Conversely, dysregulated autophagy may induce cell death, also known as type II cell death [8]. In recent decades, growing evidence has shown the involvement of autophagy in the pathogenesis of human disorders, including cancer, neurodegeneration, myopathy, aging, and cardiovascular diseases [9]. Notably, some pharmacological and nutritional modulators of autophagy have been clinically developed to treat a variety of human disorders [10]. Despite enormous progress in this field, the dual roles of autophagy in human diseases remain incompletely understood and potentially involve both desirable and undesirable outcomes. Thus, in this article, we review the context of autophagy modulatory mechanisms and their roles in cardiac physiology and heart diseases.

## 1.Autophagy: Types, Process, and Molecular Regulators

Autophagy is responsible for the turnover of abnormal cellular structures and the elimination of microorganisms (bacteria, viruses, and fungi). Three main subcategories of autophagy are divided according to their lysosome transfer channels: microautophagy, chaperone-mediated autophagy (CMA), and macroautophagy [11]. As the most typical autophagy type, macroautophagy (hereafter autophagy) indirectly delivers its goods to the lysosome via serial vesiculation, while the cargoes of CMA and microautophagy are both transported to the lysosome directly, without vesicles [12, 13]. The first morphological feature of autophagy initiation is the formation and development of a phagophore, a double-membraned autophagosome predecessor that expands its membrane to envelop substrates to shape an autophagosome. Subsequently, the autophagosome migrates along microtubules and fuses with lysosomes, leading to the formation of autolysosomes that degrade the vesicular contents. This evolutionary, highly conserved process is governed by a succession of molecules, namely autophagy-related genes (ATGs).

The ATG molecular pathway is regulated by an array of signaling molecules that respond to environmental conditions, such as calorie restriction, hormones, and the accumulation of malignant substrates that include problematic mitochondria (mitophagy), aggregated proteins (aggrephagy), excessive peroxisomes (pexophagy), and invasive pathogens (xenophagy) [14, 15]. With autophagy initiation, the first step in the formation of phagophores requires mutual interaction between class III phosphoinositide 3-kinase (PI3K-III/Vps34) and Beclin1 (Vps30/Atg6), which induces the uncoordinated-51 like autophagy activating kinase 1 (ULK1/ATG1) to interact with ATG13. During the subsequent stage, the formation of the ATG12-ATG5-ATG16L1 complex, the transformation of microtubule-associated protein 1 light chain 3 (MAP1LC3/ATG8/LC3), and a chain of ubiquitin-like reactions are required to sustain the phagophore extension. More specifically, autophagy-related E1-like enzyme ATG7 and E2-like enzyme ATG10 are critical for the ATG12-ATG5 conjugation, which thereafter can recruit ATG16L1 non-covalently to assemble the ATG12-ATG5-ATG16L1 complex to assist ATG8/LC3 recruitment and facilitate phagophore localization to the outer membrane of newborn autophagosomes. During LC3 lipidation, ATG4, a C-terminus cysteine protease, is required to process pro-LC3 to LC3-I, which further anchors to the autophagosome membrane to form LC3-II, with the participation of E1 ATG7, E2 ATG3, and E3 ATG12-ATG5-ATG16L1 (thus LC3-II is a hallmark of mature autophagosomes). In the final stage of autophagy, the process of newborn autophagosome fusion with the lysosome is called autophagic degradation or autophagic flux, which leads to the formation of a single membrane-structured autolysosome [16, 17]. These ATG molecules link ubiquitinated cargoes to the autophagosome and recruit encapsulated cargoes into the autolysosome to degrade through the activity of lysosomal lipases, nucleases, proteases, and glycosidases, and the cell then recycles the degraded products for reuse [18].

**Figure 1.**
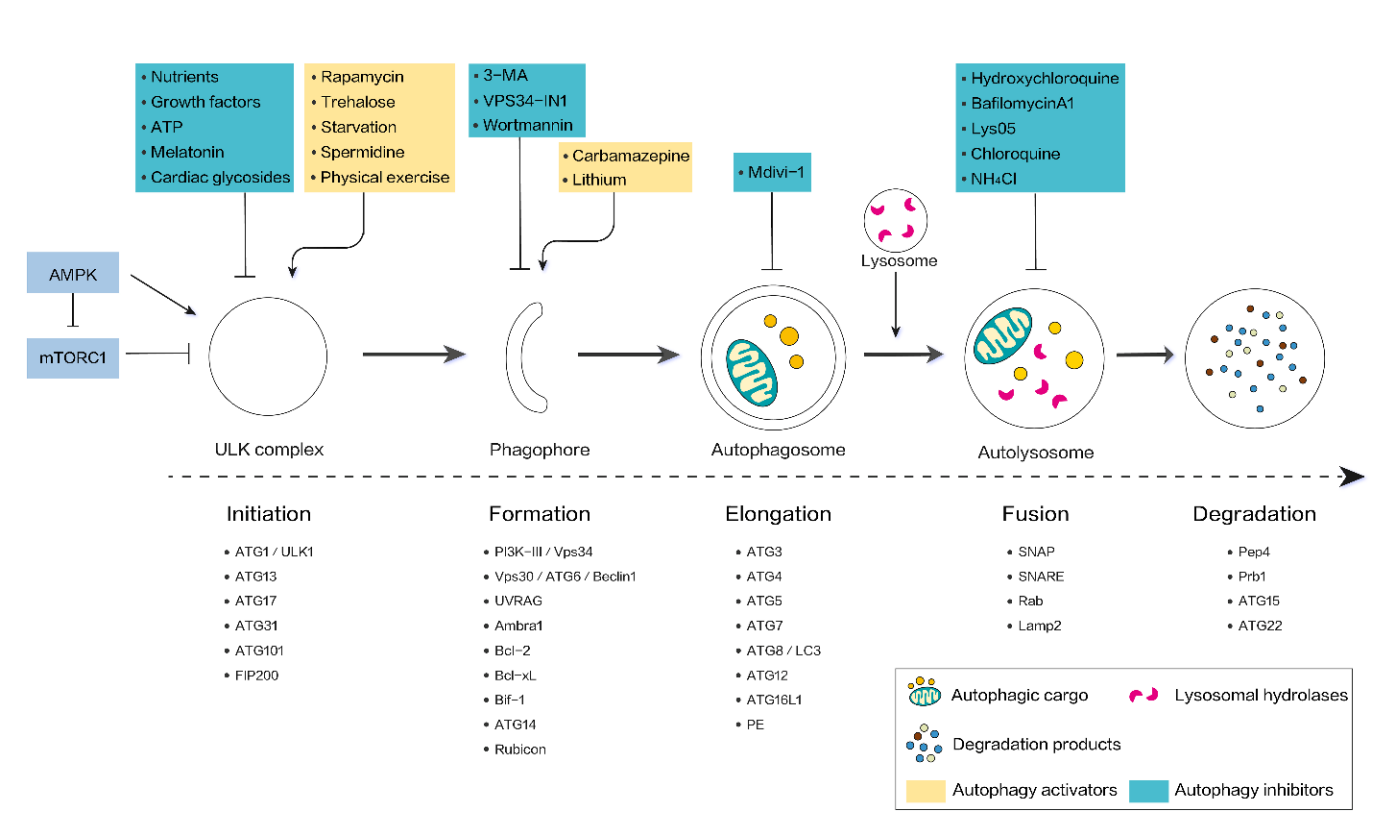
Overview of major components and modulating sites in mammalian autophagy. Autophagy can be activated by AMPK, which directly stimulate the ULK complex or indirectly stimulate through mTORC1 inhibition. Various autophagic molecules participate in subsequent formation, elongation, fusion, and ultimately, degradation. In addition, pharmacological and nutritional interventions are accessible to induce/inhibit autophagy activities through multistage validation mechanism. Abbreviations: 3-MA:, 3-methyladenine; AMPK, AMP-activated protein kinase; FIP200, focal adhesion kinase family interacting protein of 200 kDa; Mdivi-1, mitochondrial division inhibitor; mTORC1, mechanistic target of rapamycin complex 1; PE, phosphatidylethanolamine; PI3K-III, class III phosphatidylinositol-3-kinase; ULK1, UNC51- like autophagy activating kinase 1.

Among the above-mentioned autophagic proteins, the autophagy-initiating unit, the ULK complex (ULK1/ATG1, ATG13, ATG101, and FIP200), is of vital importance, as its inactivation can result in shapeless phagophores [19]. Two ULK complex regulators include: AMPK (the cell energy sensor) and mTORC1 (mTORC2 acts primarily on cellular survival and cytoskeletal organization), both of which play separate roles in regulating the ULK complex and are sensitive to cellular adenosine monophosphate (AMP)/adenosine triphosphate (ATP) ratio [20]. For instance, nutrient deprivation activates AMPK, leading to the phosphorylation of ULK1 and autophagy initiation [21]. In contrast, the presence of nutrients can activate mTORC1, suppress the ULK1 complex, and inhibit autophagy [20]. In addition to direct ULK-1 phosphorylation, AMPK also plays an inhibitory role in mTORC1 and thus contributes to the initiation of autophagy [22]. Besides, autophagy also exerts multiple functions through pharmacological and nutritional interventions (Fig. 1) and gives full access to the regulation of cellular metabolism [10, 23, 24].

## 2. Autophagy and the Heart

### 2.1. Autophagy in Physiology

In cardiac homeostasis, basal autophagy becomes important because of its housekeeping function, which is ascribed to the major cell type in heart tissue—cardiomyocyte —, which is a terminally differentiated cell and depends on autophagy for maintaining viability and functionality [25]. The link between autophagy and cardiac homeostasis in physiological settings has been extensively studied by experimentally modulating autophagy-relevant genes. *ATG5* knockout mice, since *ATG5* is necessary for optimal autophagy [26], develop cardiac hypertrophy, dilated cardiomyopathy, and contractile dysfunction with the accumulation of ubiquitinated proteins, chaotic sarcomere structures, and mitochondrial aggregation [27, 28]. Analogously, SIRT6 (sirtuin 6) is a preautophagy histone deacetylase that function in stress response, and *SIRT6-/*- mice are characterized by cardiac hypertrophy and heart failure [29, 30]. In agreement, in both humans and mice, deficiency in lysosome-associated membrane protein-2 (LAMP-2) can result in the spontaneous development of severe cardiomyopathy, referred to as Danon disease, which is accompanied by autophagic vacuolar aggregation and lysosomal function damage [31, 32]. The impaired autophagy that results upon depletion of *ATG5* can trigger serious cardiomyopathy [27]. In contrast, the promotion of autophagy upon injection of rapamycin (an autophagy inducer through mTOR inhibition) or by the induction of caloric restriction, such as through spermidine, protects against cardiomyopathy [33, 34].

### 2.2. Autophagy in Aging

The essential role of autophagy is especially emphasized during the process of cardiac aging, which requires extensive degradation of damaged proteins and organelles. The heart is an extremely hardworking organ that consumes large quantities of ATP, and thus, around 40% of the cytosolic volume in a cardiomyocyte is occupied by mitochondria, which are the major source of ROS in the cell [35]. Furthermore, mitochondria also play an important role in apoptosis modulation, Ca^2+^ release, and mitochondrial DNA (mtDNA) transcription, all of which perform key functions in the aspects of mitochondrial-quality control and the ERS response [36]. In the aging process, metabolic and hormonal changes may contribute to lower levels of autophagy, which results in giant dysfunctional mitochondria that exceed the engulfment capacity of autophagosomes [37, 38]. Enlarged mitochondria typically exhibit reduced respiratory function, decreased ATP production, and increased ROS generation [39]. ROS, in turn, can compromise mitochondrial proteins and mtDNA; hence, the vicious cycle starts and continues [40]. Of note, the induction of autophagy by caloric restriction has been shown to attenuate mitochondrial impairment, reverse cardiac hypertrophy, restore diastolic dysfunction, and improve cardiovascular fitness in aging animals [41, 42]. Rapamycin treatment in aged mice has been proven to persistently delay the development of both cardiac hypertrophy and myocardial stiffness and alter the activity of mitochondrial respiratory chains [43]. Moreover, the depolarization of mitochondrial membrane potential (ΔΨM) has previously been shown to be associated with enhanced levels of ROS in myocardial cells [44]. In 2006, Schieke et. al. demonstrated that rapamycin could reduce ΔΨM from a higher level to a more physiological level [45], which suggests that rapamycin might lower the electron flux in the respiratory chain, thus leading to a decline in ROS generation, with subsequent beneficial effects on the aging heart in the following process. Nevertheless, in AMPKa2 KO mice, the protective effects of caloric restriction and rapamycin on cardiovascular homeostasis seem to be abolished during aging [46, 47]. Collectively, these studies suggest that the basal autophagic response might be an important process that limits physiological decline during aging, as the impairment of autophagy worsens both cardiac function and morphology.

### 2.3. Autophagy in Diseases

While the crucial role of basic autophagy in cardiac aging is well established, stress-activated autophagy in cardiac diseases is more complex, poorly understood, and likely dependent on the severity and duration of the insults. During ischemic events in myocardial infarction (MI), autophagy is rapidly upregulated [48], and the level of autophagy is inversely correlated with that of apoptosis [49], which suggests that autophagy could prevent cell death under an acute coronary ischemia attack. Deletion of Mst1 activates autophagy and alleviates cardiac remodeling in the heart after MI [50]. However, autophagy appears to be detrimental during heart reperfusion after ischemia. Increased levels of ROS can enhance autophagosome formation and autophagic flux through the upregulation of Beclin1 in the process of myocardial reperfusion injury [51]. Furthermore, the results of Matsui et. al. showed an apparent decrease in infarct size in Beclin1(+/-) mice after ischemia/reperfusion (I/R) myocardial injury [52]. In an I/R mouse model, inhibition of autophagy was found to protect the heart from I/R injury by activating the Pink1/FAM65B pathway, which further indicates that autophagy can promote cell death during reperfusion [53]. Interestingly, autophagy stimulated by ischemia relies on an AMPK-dependent mechanism, whereas autophagy stimulated by reperfusion seems to act in an AMPK-independent but Beclin1-dependent manner [54], which implies that there may be differences between ischemia and reperfusion with respect to their underlying autophagy mechanisms. It must be noted that the induction of autophagy may also attenuate I/R myocardial injury [55], and this could be partly explained by the recycling of amino acids and fatty acids from dysfunctional cytosolic components, which provide a compensatory catabolic energy source during hypoxia and starvation during ischemic insults.

The autophagy activity of cardiomyocytes is context-dependent during heart failure (HF). In animals bearing thoracic aortic constriction (TAC) [56, 57] or phenylephrine [57], suppression of autophagy is observed in the initial stage of cardiac hypertrophy, whereas autophagy is activated during the subsequent development of HF. Initially, the pharmacological induction of autophagy with rapamycin was shown to improve ventricular function in mice with decompensated cardiac hypertrophy [58]. In this context, completely disrupting autophagy can rapidly lead to HF [59]. It seems reasonable to suppose that a modest activation of autophagy is required to maintain cardiac function during the process of hypertrophy in hearts subjected to moderate pressure overload (PO). On the other hand, in PO-induced HF, an excessive induction of autophagy may trigger cardiomyocyte death in the failing heart [60]. Advanced glycation end products (RAGE) are involved in the progression of HF by upregulating autophagy. In TAC mice, RAGE knockout or blockade has been demonstrated to downregulate autophagy-related proteins and reduce cell death after TAC. The death of ventricular myocytes triggered by RAGE is also alleviated by ATG5 knockdown [61]. Thus, in the severe-PO context, autophagy activation should be reduced, because excessive autophagy might degrade key proteins and mitochondria, both of which are vital for cell survival [62]. Compared to the wild-type (WT) group, cardiomyocyte autophagy and ventricular remodeling induced by severe PO both significantly decreased in Beclin1(+/-) mice [56]. In this study, the overexpression of Beclin1 that was elicited by the cardiac-specific α-MHC promoter in Tg mice could broaden autophagy activity and boost pathological remodeling in the heart. Conversely, trichosatin A has therapeutic effects in inhibiting autophagy and blunting ventricular remodeling in a severe-PO context; thus, it may be a valid remedial tool to delay or even reverse pathological remodeling [63].

To summarize, under baseline condition, the steady state of the heart requires a low level of autophagy. In acute pathological circumstances where more autophagy is needed, up-regulating autophagy is necessary to eliminate protein aggregates (protein toxic stress, hypertrophy) or damaged organelles (aging, ischemia). In the case of intensive and prolonged stress, maladaptive autophagy is observed, which can lead to pathological progression [64-66]. Basal autophagy may be effective in the heart; however, the overexpression of autophagy can be either beneficial or detrimental in the context of cardiovascular diseases.

## 3. Convergence of Autophagy and CAVD

Since the 1960s, autophagy has attracted the attention in the study of human pathologies, such as cardiovascular disorders [67]. Recently, there has been renewed interest in the relationship between autophagy and CAVD [68]. During aging, diminished autophagy is related to various malfunctions, including the accumulation of misfolded proteins [69], lipid oxidation by-products [70], and impaired macromolecules [71], and is hypothesized to be caused by the age-related decrease in lysosomal enzymes activity [72]. Interestingly, CAVD manifests as an age-related progressive disorder, and epidemiological studies support the link between aging and CAVD [73]. Thus, it is plausible that there might be an intimate link between CAVD and autophagy.

To date, the role of autophagy has been unfolded in diverse paramount cardiac pathologies, including left ventricular hypertrophy [74], cardiomyopathies [75, 76], and ischemic injury of the heart [77]. Despite extensive studies, however, the mechanisms responsible for the effects of autophagy on valvular diseases remain scarce, and no definitive results are available. In this work, evidence is presented to implicate the action of autophagy in CAVD and its dual roles during pathological progression.

### 3.1. Aortic Valve Structure: From Histological Integrity to Histopathology Heterogeneity During CAVD

Normally, aortic valves regulate flow from the ventricle into systemic circulation with the coordination of three membrane structures, called leaflets or cusps. This on-off function of the valve leaflet requires microarchitecture integrity in three layers: fibrosa, spongiosa, and ventricularis [78]. The ventricularis, which faces the ventricular outflow tract, is mainly composed of elastic fibers that allow the leaflets to stretch in diastole and rebound in systole. The spongiosa, the central layer, is largely comprised of glycosaminoglycans that function as shock absorbers during cyclical valve motion. The fibrosa, on the aortic side, is rich in collagen bundles and provides suitable hardness and stiffness during diastole [78]. Interestingly, one of the paramount features in CAVD progression is the growing deposition of misaligned collagen, which predominantly emerges in the fibrosa layer of the leaflets and results in a dissymmetry histopathology. Collagen produced by valve interstitial cells (VICs) generally acts as a scaffold, but excessive collagen production causes disorganization and stiffness of the leaflet, with a decrease in biodynamic function [79]. Other extracellular matrices such as tenascin-C, a glycoprotein involved in cell proliferation and differentiation, and biglycan, a proteoglycan implicated in the osteogenic response, can be progressively deposited during fibrosis of the aortic valve [80, 81].

At the cellular level, homeostasis of the aortic valve leaflet is regulated by two resident cell populations: aortic valvular endothelial cells (AVECs) and aortic valvular interstitial cells (AVICs). CAVD typically manifests itself in AVEC dysfunction and heterogeneous differentiation of AVICs [82]. AVICs, the most galore population residing within all the layers of the valve that directly remodels the extracellular matrix [83], originate from endothelial-to-mesenchymal transition (EndMT) and are preserved by hematopoietic stem cell (HSC) contributions [84, 85]. The AVIC population harbors a high degree of phenotypic plasticity, with different functional types in the resting and diseased states. In a quiescent state, AVIC is described as a fibroblast-like phenotype in nature, whereas activated AVIC can differentiate into a myofibroblast phenotype or undergo osteogenic differentiation in CAVD [86]. Transformation of AVICs to the myofibroblast phenotype is believed to be responsible for the fibrotic accumulation of collagen during the initial stage of CAVD [87]. The potential contributors to the myofibroblast phenotype change include mechanical stretch and transforming growth factor-β (TGF-β), both of which play a synergetic role in AVIC synthetic activity, collagen accumulation, and extracellular matrix remodeling [88, 89]. AVICs show great sensitivity to mechanical stretching and selectively undergo phenotypic changes based on specific mechanical environments. In detail, at a stiffness level of approximately 15 kPa, AVICs can perform myofibroblastic activation along with apparent expression of alpha smooth muscle actin (αSMA)-positive fibers (αSMA is regarded as an indicator of myofibroblasts) [90], whereas mechanically challenged AVICs can experience osteogenic-like differentiation under 25 kPa [91]. Notably, the results from Quinlan et. al. [92], and Kloxin et. al. [90] showed that activated AVICs can be deactivated to a quiescent state by decreasing the matrix stiffness below 10 kPa. In addition, myofibroblast activation can also be triggered by profibrotic cytokines, especially TGF-β1, which can potentiate AVIC differentiation into myofibroblasts and increase αSMA expression [83]. TGF-β1 mediates myofibroblast activation by activating Smad transcription factors [93], upregulating matrix metalloproteinase 2 (MMP-2) expression [94] and stimulating the p38/Egr-1 pathway [95]. Importantly, AVICs subjected to mechanical insults become hypersensitive to TGF-β, and this implies that mechanical environments can modulate the effects of TGF-β [92]. Notably, TGF-β1 fails to induce myofibroblast differentiation in soft substrates [96]. In addition, VIC undergoes osteoblast-phenotype changes upon microenvironment alteration, such as ROS and inflammatory cytokines, with osteogenic nodules observed in the affected leaflets. Growth factors such as bone morphogenetic protein-2 (BMP-2), an inducer of osteoblast differentiation that is implicated in CAVD progression, are thought to promote VIC osteogenic-reprogramming and lead to the elevation of pro-osteogenic activity in valvular calcification [97, 98]. BMP-2 is a potent osteogenic morphogen that is implicated in the regulation of bone development. In human stenotic valves that are obtained during surgical excision, BMP-2 expression has been detected in T/B-lymphocyte-rich areas where ossification is identified, although BMP-2 is modestly detected in control valves [99, 100]. In human cultured AVICs, BMP-2 induces osteoblast differentiation by upregulating alkaline phosphatase (ALP) activity [98]. This activated phenotype might be responsible for the osteogenic nodules observed in CAVD valve leaflets [101].

**Figure 2.**
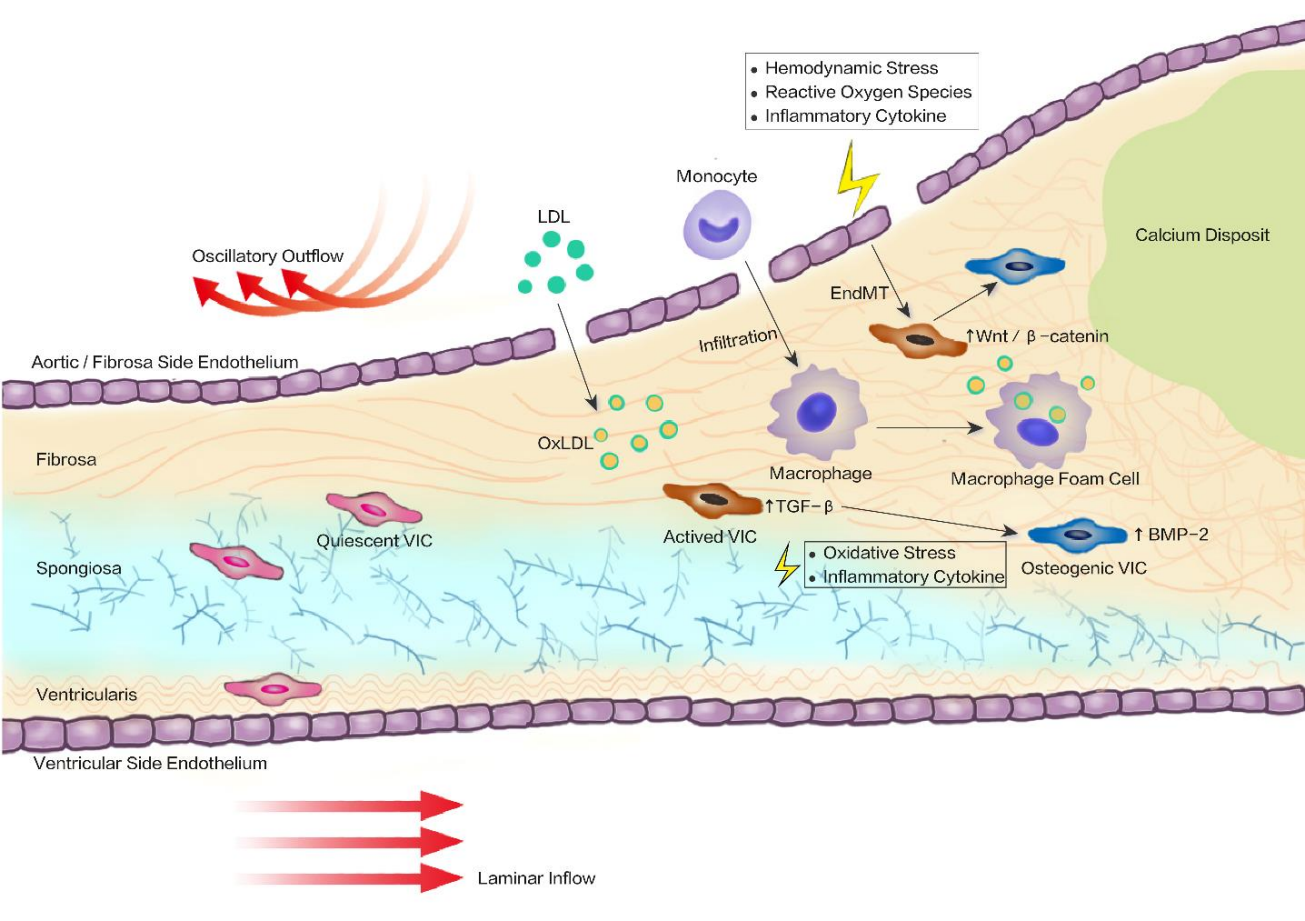
Schematic presentation of calcified aortic valve progression. The structure of the aortic valve leaflet consists of three-layer extracellular matrix (ECM): the fibrosa, the spongiosa, and the ventricularis; with valvular interstitial cells (VIC) scattering in all three layers and a monolayer of valve endothelial cells (VEC) overlaying in both sides. Blood flow (haemodynamics) in ventricular side is steady-going and laminar, whereas is oscillatory and mussy in fibrosa side, from which the lesion is vulnerable to initiate. Endothelial damage can be triggered by several factors including hemodynamic stress, reactive oxygen species, and inflammatory cytokine, followed by lipid accumulation, inflammation infiltration, cell phenotype transformation, and extracellular matrix remodeling. Finally, fibrotic remodeling and valve mineralisation lead to leaflet thickness and valve dysfunction. Abbreviations: BMP-2, bone morphogenetic protein-2; EndMT, endothelial-to-mesenchymal transition; LDL, low-density lipoprotein; OxLDL, oxidized LDL; TGF-β, transforming growth factor- β; VIC, valve interstitial cell.

In addition to AVICs, AVECs encapsulate the valvular blood-contacting surfaces and line up to form a block to separate valve tissue components from the circulating blood. Nevertheless, the control of valve homeostasis by AVECs might go beyond serving as a barrier. Valve pathology has been proven to originate from systemic endothelial dysfunction, possibly due to the ability of endothelial cells to perceive and react to mechanical or biomedical signals [102, 103]. For example, during CAVD, AVECs progressively invade the leaflet interstitium and undergo a phenotypic and functional transition to mesenchymal or fibroblast-like cells (also known as the EndMT process), which has been shown to respond to shear stress and Wnt/β-catenin signaling stimulation [104]. In EndMT, AVECs lose endothelial markers, such as CD31, CD144, von Willebrand factor (vWF), and platelet-derived growth factor (PDGF), but regain mesenchymal or myofibroblastic markers, such as αSMA, MMP-2, and collagen types I and III, all of which are observed in myofibroblast activation of AVICs [105, 106]. Numerous studies have highlighted the role of EndMT in cardiovascular diseases, including atherosclerosis, pulmonary arterial hypertension, graft vessel restenosis after coronary artery bypass grafting (CABG), and myocardial infarction [107]. EndMT in valvular settings seems to lack intensive and profound studies. However, the EndMT process is crucial for both embryonic and adult valve remodeling [108]. As for EndMT in CAVD, AVECs undergo osteogenic differentiation via a TGF-β1-mediated EndMT process, with apparent osteogenic changes confirmed by increasing mRNA levels of osteocalcin, osteopontin, and Runx2 [109]. Intriguingly, the above osteogenic differentiation of AVECs could be reversed by AVICs, implying that AVICs closely interact with AVECs and inhibit the EndMT and osteogenesis process of AVECs [109]. Of note, AVECs exhibit side-dependent phenotypic properties [110, 111], and this inconformity may be due to the fluid flow profiles: the inflow (ventricularis) side VECs are exposed to steady, laminar shear flows, whereas the outflow (fibrosa) side VECs are indwelt in oscillatory and turbulent flows; thus, the fibrosa-side VECs are prone to hemodynamic and metabolic offenses, along with inflammation infiltration, lipid accumulation, and cell phenotype switching. Moreover, the oscillatory shear-exposed fibrosa layer has been shown to initiate calcific degeneration in vivo, which may result in typic pathologic remodeling in the fibrosa side of the valve [112] (Fig. 2).

### 3.2. Detection of Autophagy in CAVD

Detection of tissue autophagy is currently a poorly developed field. Even though the related detection guidelines have recently been set up [113], the ideal method of detecting autophagy in calcific valves does not yet exist. Therefore, verifying the autophagy responses with diverse means is recommended as follows: morphological identification by electron microscopy, together with autophagic marker (like LC3 or ubiquitin) detection and quantificational determination via western blotting or fluorescence microscopy. Morphologically, the identification of autophagy by transmission electron microscopy (TEM) is the most commonly applied approach to evaluate autophagy [113]. Ultrastructurally, abundant autophagic vacuoles followed by well-preserved organelles can be observed under TEM in cultured AVICs, and these features suggest that the functionality of AVICs is in good shape under basal autophagy flux [114]. Of note, potential pitfalls should be considered, while there might be some misinterpretations in distinguishing autophagosomes from other cellular components [115]. Namely, researchers may misjudge swollen mitochondria as autophagosomes or incorrectly refer to lipid droplets as autophagic vacuoles. Given that TEM identifies autophagy vacuoles with many drawbacks, non-TEM-based techniques, such as marking autophagy in tissue, can provide advantageous alternatives (e.g., analysis of GFP-LC3 dots by fluorescence microscopy, LC3 lipidation on a western blot, or LC3 and ubiquitin detected by direct immunohistochemical techniques) [116, 117]. Nevertheless, in contrast to LC3, evaluating autophagy levels by quantitatively marking ubiquitin remains an unexpected pitfall. More specifically, quantitative analyses of autophagy in human AVICs with LC3 and ubiquitin indicate that ubiquitin puncta are relatively small, and there are often no statistically significant differences between normal AVIC, diseased AVIC, and ATG7-knockdown AVIC samples, whereas LC3 can show significant differences between normal, disease, and ATG7-knockdown conditions [118]. This paradox may result from the “out-of-step” phenomenon between ubiquitin formation and autophagic activity. For instance, ubiquitinated aggregation could appear in ATG7-knockdown mice despite impaired autophagy function [119]. This may be because, in addition to excessive autophagy activity, ubiquitinated aggregation can also derive from inhibited autophagy and the accumulation of misfolded proteins [120]. Hence, intracellular morphometrics by TEM together with LC3 quantitative analysis provides a preferred method for quantifying autophagy in both aortic valve and other tissues.

### 3.3. Protective effects of Autophagy in CAVD

CAVD is an active atheroinflammatory disorder, rather than a passive degenerative disease, and is associated with progressive mineralization of the leaflets without rheumatic heart disease [1]. The multifactorial pathogenesis of CAVD includes, but not confined to, chronic inflammation [121], lipoprotein deposition [122], and active calcification [123, 124]. In particular, maladaptive autophagy plays an evident part in pathogenesis of several cardiovascular-related pathologies such as ischemic heart disease [125], heart failure [126], stroke [127], and diabetes [128]. Thus, the researchers wonder if autophagy is associated with valvular diseases in heart? Whether autophagy in CAVD serves as a protector like it acting in basal autophagic flow, or it shifts from a protector to a poisoner during disease progression?

**Figure 3.**
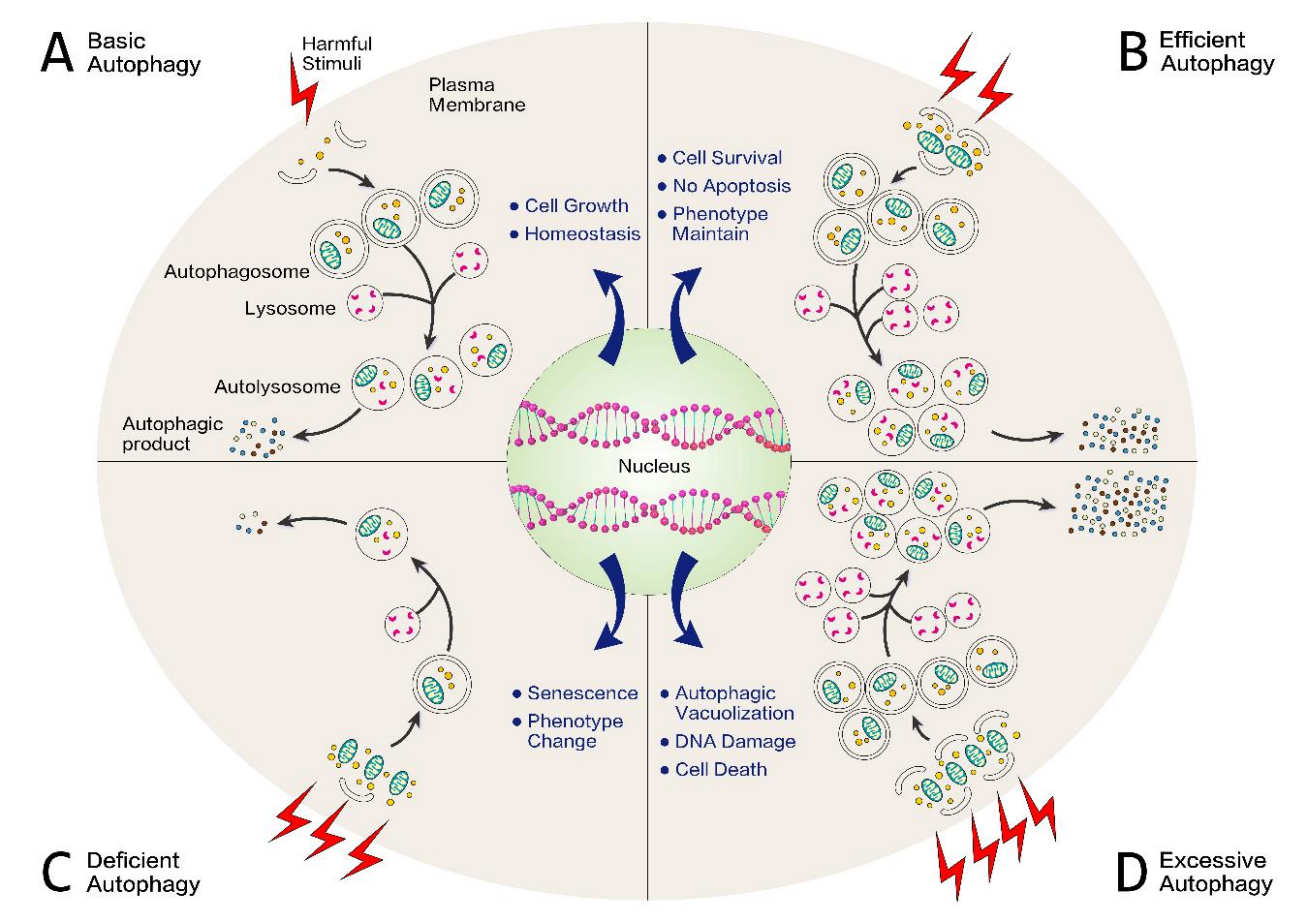
Protective versus detrimental role of autophagy in CAVD. Upon stimuli, cell undergoes different events depending on the grades of the impairment and the sensitivity of the cell: Basal autophagy defends cell against toxic molecules which result from harmful stimuli insult via the lysosomal-degrading pathway, thus cell grows and remains homeostasis (A). Stimulating autophagy in relatively mild perturbation setting is expected to hasten the removal of damaged components and favors phenotype maintenance and survival (B). In response to very harsh environmental condition, autophagy is not sufficient for the removal of cellular damage, followed by phenotype alteration and senescence (C). Under a long-term chronic state of stimuli, severe oxidative stress combined with excessive autophagic flux may also lead to DNA damage and promote cell death (D).

The first reported histological evaluation of autophagy in calcified aortic valves took place in 2006, when Somers et al. highlighted the presence of autophagy in calcified aortic valves (noticeable ubiquitin labeling in 12 calcifications of aortic valves and negligible labeling in 12 control valves) [129]. Conversely, in 2017, Deng et al. reported an LC3-based immunohistochemistry study, in which AVICs in donors with calcified valves displayed significantly lower autophagic activity than in healthy donors [118]. This discrepancy may be attributed to three possible causes: 1) drawbacks in using ubiquitin versus LC3 as a marker of autophagy (discussed in Section 3.2); 2) in Somers’ study, the investigators histologically evaluated autophagy on the whole valve tissue, in contrast to Deng’s investigations of AVICs alone; 3) during disease progression, autophagy can switch from an active to an impaired state.

Among all tissues, the basal autophagy level is relatively high in the whole aortic valve [130] and in AVICs [114, 118]. This may be caused by the aortic valve, as this is a portion of the cardiovascular system that is subjected to continuous environmental insults, including metabolic and mechanical stress. As a result, its normal function could be relatively highly dependent on autophagy, the rate of which can change rapidly and dramatically in response to different stimuli (Fig. 3A) [131, 132]. Recently, vascular autophagy has been shown to act as a safeguard mechanism against calcification. Specifically, pharmacological inhibition of autophagy aggravates the calcification of vascular smooth muscle cells (VSMCs), whereas the induction of autophagy attenuates VSMC calcification [133]. In line with this finding, suppressing autophagy in human AVICs greatly induces pro-osteogenic activity followed by remarkable calcium deposition, and autophagy stimulation notably decreases the osteogenic response, which serves as a protective and pro-survival role in the calcification process (Figure 3B, 3C) [118]. In this study, suppression of autophagy by two chemical inhibitors (3-methyladenine/3-MA and bafilomycin/BAF) or by genetic knockdown of Atg7 augmented the expression of pro-osteogenic biomarkers BMP-2 and ALP, followed by aggravation of ALP activity and calcium deposition in normal AVICs. On the other hand, such an osteogenic response was weakened by the activation of autophagy triggered by rapamycin or by genetic upregulation of the *ATG7* gene.

Greater insights into autophagy events have been provided by studies that investigated autophagy using pharmacological manipulation during CAVD. For example, signal transducer and activator of transcription 3 (Stat3) possesses anti-inflammatory effects and has been demonstrated to induce a series of cardioprotective functions, such as reduction of infarct size, improvement of cardiac function, and decrease of myocardial apoptosis [134]. In VICs from the human aortic valve, toll-like receptor 4 (TLR4) acts as a proinflammatory factor. Furthermore, TLR4-driven inflammation is negatively regulated by Stat3, and Stat3 inhibition exacerbates inflammation and calcification [135, 136]. Rapamycin, a drug commonly used for immunosuppression and cancer treatment, decreases TLR4-mediated inflammation in human AVICs by triggering the Stat3 signaling pathway with autophagy involvement, consequently serving an anti-inflammatory role in CAVD [137]. Moreover, autophagy, which regulates the inflammatory cell homeostasis and cytokine secretion, plays a key role in the inflammatory process. IL-1β is an important proinflammatory cytokine that is secreted by immune cells and triggers inflammation. Inhibition of autophagy by treating macrophages with 3-MA promotes IL-1β secretion, whereas the further induction of autophagy with rapamycin inhibits IL-1β secretion [138]. Thus, the autophagy protein quality control system provides a basic compensatory and pro-survival mechanism for valvular cells to endure pathological offenses, especially since reduced autophagy could make AVIC inactive, which might spur VIC death [130]. Notably, AVIC viability can preserved through the activation of autophagy. It has been demonstrated that trehalose, the latest cryoprotectant for tissue cryopreservation, maintains the viability of allograft valves by promoting autophagy against apoptosis via the p38-MAPK signaling pathway [139]. The valves of cryoprotected rats treated with trehalose exhibit lower expression levels of glucose-regulated protein 78 (GRP78), CCAAT/enhancer-binding protein homologous protein (CHOP), caspase-3, and p62, but higher levels of LC3, Atg5, and Atg7, which indicates that trehalose-activated autophagy suppresses ERS-induced apoptosis. Chang et. al. also confirmed the cryoprotective effect of trehalose on aortic valve grafts [140], and such an autophagic agent may provide promising prospects for the development of noncalcifiable bioprosthetic valves with reliable functionality and durable bioactivity.

The most striking feature of the CAVD process is valve fibrosis and calcification, and such osteogenic activity seems to be associated with pro-calcifying conditions, such as elevated blood phosphate (Pi) levels [141]. Human AVICs display calcium deposition induced by long-term exposure to high blood Pi levels [142]. Interestingly, high Pi-treated AVICs show a significant increase in LC3-II levels, and increased autophagy in AVICs is well recognized as a survival mechanism and might occur before calcification [130]. Moreover, autophagy activity in AVICs seems to be affected by Pi concentration [114]. Specifically, AVICs treated with low/middle Pi concentrations (0.8 mM/1.3 mM Pi) underwent atypical autophagic progress, in which organelles were sequestered and digested through the giant endoplasmic reticulum (ER), rather than through lysosomes in an orthodox autophagic manner. Paradoxically, however, AVICs treated with upper-normal Pi concentration (2.0 mM Pi) presented peculiar degeneration (non-apoptotic, lipid-release-associated cell death and mineralization), which had been previously described in *vitro* models in both dystrophic- and metastatic-like hyperphosphate conditions [143, 144]. Notably, compared with orthodox autophagy, the role of this non-lysosomal, ER-dependent autophagic activity in guarding AVICs against cell death and subsequent mineralization, to some extent, may represent a time-saving process in stressed cells that restores homeostasis more quickly and allows different cell fates upon exposure to pathological insults. In addition, AVICs from donors with calcification show considerably lower autophagic activity than those from healthy donors [118], which supports the concept that salvage effects of autophagy in CAVD may be abolished by high and prolonged insults, just as in other diseases [13, 145-147].

### 3.4. Autophagy from Survival to Death in CAVD

In addition to its protective properties, autophagy can also be associated with cell death mechanisms [8]. Morphological studies using electron microscopy have historically classified three types of death: Type Ⅰ apoptosis, Type Ⅱ autophagic cell death (which is characterized by massive intracellular autophagic vacuoles), and Type Ⅲ necrosis [8]. It is now increasingly accepted that apoptosis and necrosis may not be the exclusive mechanisms of cell death, but autophagy may be an alternative final fate of various cardiovascular diseases, such as heart failure [148], non-ischemic cardiomyopathy [149], ischemic heart disease [150], and atherosclerosis (which is a twin disease to CAVD) [138]. In addition, evidence has shown that, unlike basal autophagy, excessive autophagy might induce cell death, which represents a primary mechanism of leaking toxic substances in degenerative aortic valves, thereby attracting inflammatory cells and ultimately incurring mineralization (Figure 3D) [151]. However, while several clues suggest that autophagic cell death, rather than apoptosis, is the dominant factor associated with inflammation infiltration in CAVD [120, 129, 151, 152], other paradoxes illustrate that apoptosis can be observed in the vicinity of calcified regions [153-156]. To further complicate this matter, autophagy and apoptosis, both manifested as the mechanisms of programmed cell death (PCD), are not mutually exclusive pathways but are frequently blended and complicated by the same stimulation, share common signaling pathways, and are subjected to complex crosstalk [157]. The paradox between autophagy and apoptosis in calcific aortic leaflets may be due to tangled crosstalk, as autophagy can either antagonize, favor, or occur in parallel with apoptosis. More interestingly, although Jian et al., reported that apoptosis exclusively occurs in cultured sheep AVICs with calcification [158], other studies showed that neither typical apoptosis nor necrosis features were ultrastructurally or immunopositively encountered; rather, particular traits of autophagy included myelin figures and massive vacuolation, coupled with lipid-release-associated procalcific cell death [114, 143, 144]. Such degenerative features could, in a way, deepen the notion that autophagic cell death, as previously reported [129], might be primed for calcified aortic valve stenosis; but still, future investigations need to figure out whether the procalcific degeneration pattern is a type of sharply unexpected cell collapse or a well-organized action of autophagic cell death.

The interplay between autophagy and cell death can be affected by several factors, including the extension of autophagy and microenvironmental conditions [159]. The initiation of cell death can be caused by inflammatory cell infiltration or oxidized lipid accumulation, which have previously been exhibited in atherosclerosis as well as CAVD, especially around the procalcific region [160-163]. For example, evidence in human aortic SMCs reveals that cytotoxic oxLDL oxysterol can activate oxidative stress-induced cell death via the induction of autophagy [164-166]. The activation of autophagy in SMCs is generally considered to be propitious to cell survival and plaque stability, but excessive autophagy may lead to SMC death, thus resulting in plaque destabilization and rupture, followed by thrombosis and acute clinical events [138]. Moreover, a hallmark of CAVD progression is the inflammatory infiltration of macrophages, which subsequently turn into foam cells with oxLDL-filled cytoplasm [167]. Recently, the results from Yamada et. al. showed that autophagic markers co-localize with macrophage-derived foam cells in valves with atherosclerotic involvement [152]. However, no further evidence has revealed the relations between autophagy and cell death, although everolimus (autophagy inducer)-eluting stents implanted in atherosclerotic arteries may selectively induce the death of macrophages [168]. Additionally, it is well known that autophagy can protect endothelial cells (ECs) from harmful stimuli such as oxLDL [169] and ROS [170]. Nevertheless, under conditions of oxidative stress, autophagy in ECs may also shift from a defensive to a cell-death promoting event [171], which is similar to the process of betides in macrophages and SMCs.

Aside from lipid accumulation and inflammatory infiltration, loss of anti-calcific protein, osteogenic phenotype shift, and mechanical and metabolic effects can also contribute to calcium deposition in aortic valves [172, 173]. This is at least in part similar to the processes that occur in atherosclerosis, during which autophagy is closely involved in the formation of atherosclerotic plaques [174, 175]. Nonetheless, probes are required to further elucidate the factors that stimulate autophagy in CAVD.

## 4. Concluding Remarks

Since the 1960s, autophagy has been extensively explored in human disorders, such as neurodegenerative diseases, acute kidney injury, chronic lung disease, cardiovascular disease, and cancer. Surprisingly, the dysregulation of autophagy in CAVD has only recently been addressed. In addition, it is ill-defined whether autophagy is a foe or friend of CAVD. Most probably, basal autophagy aims at cell survival and homeostasis, while stress-induced autophagy might antagonize/induce cell death, depending upon the mode and intensity of the stress, as well as specific cells and involved signaling pathways. Altogether, we propose the idea of autophagy-inducing applications, but caution is still warranted, due to unexpected cell-type-dependent effects and cell death implicated with excessive autophagy; and therefore, suitably recommended doses are advised. AMPK activators, mTOR suppressors, and trehalose have been demonstrated to be effective in animal models of neurodegenerative disorders, such as Alzheimer’s, Parkinson’s, and Huntington’s diseases. These kinds of drugs are also appropriate and beneficial for the treatment of cardiomyopathies, and the available cellular experimental evidence supports a similar treatment effect on autophagy in CAVD. Finally, we believe that proper utilization of pro-autophagic agents can have far-reaching therapeutic benefits, not only for CAVD but also for other aging-related diseases; however, additional research is needed before the drugs can be used clinically.
